# Supplementary material for: Sex-biased expression of microRNAs in Drosophila melanogaster
Source: Open Biol. 2014 Apr 2;4(4):140024. doi: 10.1098/rsob.140024 (PMC4043116; doi:10.1098/rsob.140024)
Supplement: Supplementary Table 1 [file rsob140024supp1.pdf]

Supplementary Table 1. Mature microRNAs and expression fold change between sexes in *Drosophila*. Statistical values from edgeR output (see main text for details).

MicroRNA	logFC	logCPM	PValue	QValue (FDR)
dme-mir-989-3p	8.702	14.034	6.96E-090	3.04E-087
dme-mir-994-5p	8.706	13.870	3.94E-083	8.61E-081
dme-mir-318-3p	8.209	14.584	9.94E-056	1.45E-053
dme-mir-92a-3p	5.678	11.848	2.07E-053	2.26E-051
dme-mir-994-3p	8.215	9.471	6.11E-047	5.34E-045
dme-mir-977-3p	-5.004	10.206	7.56E-037	5.50E-035
dme-mir-313-5p	5.073	9.974	3.64E-036	2.27E-034
dme-mir-312-3p	4.296	11.306	5.83E-033	3.19E-031
dme-mir-311-3p	3.871	11.334	5.61E-027	2.73E-025
dme-mir-989-5p	9.843	7.363	3.55E-024	1.55E-022
dme-mir-92b-3p	4.968	10.120	1.25E-022	4.96E-021
dme-mir-310-3p	3.045	11.579	5.91E-020	2.15E-018
dme-mir-995-3p	2.942	10.824	3.11E-019	1.04E-017
dme-mir-992-3p	-4.806	8.032	5.41E-019	1.69E-017
dme-mir-318-5p	6.727	7.163	7.53E-017	2.19E-015
dme-mir-9c-5p	2.458	12.207	5.83E-016	1.59E-014
dme-mir-984-5p	-3.425	8.707	1.33E-015	3.41E-014
dme-mir-975-5p	-5.782	7.111	1.98E-014	4.82E-013
dme-mir-313-3p	3.223	8.058	2.55E-014	5.87E-013
dme-mir-985-3p	-8.834	6.552	1.11E-013	2.42E-012
dme-mir-995-5p	3.420	6.854	1.42E-012	2.96E-011
dme-mir-997-5p	-5.456	6.821	1.52E-012	3.02E-011
dme-mir-982-5p	-5.711	6.216	5.77E-012	1.10E-010
dme-mir-978-3p	-4.663	6.960	2.04E-011	3.72E-010
dme-mir-973-5p	-5.896	6.395	2.29E-011	4.01E-010
dme-mir-963-5p	-2.986	7.703	3.42E-011	5.75E-010
dme-mir-92a-5p	2.204	9.076	4.78E-011	7.74E-010
dme-mir-312-5p	2.252	8.325	2.77E-010	4.32E-009
dme-mir-964-5p	-2.754	8.086	4.07E-010	6.13E-009
dme-mir-9b-5p	2.421	12.594	5.67E-010	8.26E-009
dme-mir-998-3p	1.923	9.427	1.30E-009	1.83E-008
dme-mir-9c-3p	2.471	8.027	1.48E-009	2.02E-008
dme-mir-959-3p	-3.233	7.343	3.45E-009	4.57E-008
dme-mir-310-5p	7.830	5.374	4.44E-009	5.70E-008

dme-mir-79-5p	2.929	6.422	4.74E-009	5.92E-008
dme-mir-959-5p	-4.495	5.928	5.76E-009	6.99E-008
dme-mir-9b-3p	2.225	8.550	1.13E-008	1.33E-007
dme-mir-961-3p	-5.101	5.690	1.55E-008	1.78E-007
dme-mir-972-3p	-5.306	5.930	6.02E-008	6.75E-007
dme-mir-976-3p	-7.679	5.562	9.53E-008	1.04E-006
dme-mir-303-5p	-4.116	6.119	1.33E-007	1.42E-006
dme-mir-960-5p	-2.506	7.239	1.39E-007	1.44E-006
dme-mir-279-5p	2.986	6.034	3.37E-007	3.43E-006
dme-mir-92b-5p	3.587	5.580	5.49E-007	5.45E-006
dme-mir-79-3p	1.415	11.190	8.91E-007	8.65E-006
dme-mir-977-5p	-7.150	5.088	2.41E-006	2.29E-005
dme-mir-960-3p	-4.194	5.749	3.40E-006	3.17E-005
dme-mir-991-3p	-7.164	5.174	4.46E-006	4.06E-005
dme-mir-306-5p	1.564	10.016	9.03E-006	8.05E-005
dme-mir-12-5p	-1.139	15.865	5.36E-005	4.68E-004
dme-mir-993-3p	-1.282	10.740	5.81E-005	4.98E-004
dme-mir-996-5p	1.253	9.441	1.07E-004	9.00E-004
dme-mir-978-5p	-6.784	4.894	1.13E-004	9.31E-004
dme-mir-996-3p	1.048	13.363	1.79E-004	1.45E-003
dme-mir-184-3p	1.024	14.257	2.06E-004	1.63E-003
dme-mir-2a-1-5p	1.795	6.171	3.03E-004	2.37E-003
dme-mir-13b-2-5p	1.705	6.733	3.29E-004	2.52E-003
dme-mir-308-5p	1.129	8.890	4.62E-004	3.48E-003
dme-mir-311-5p	3.423	4.964	4.85E-004	3.59E-003
dme-mir-961-5p	-4.138	4.979	4.95E-004	3.61E-003
dme-mir-4966-5p	-6.583	4.788	7.19E-004	5.15E-003
dme-mir-314-5p	1.687	5.926	9.29E-004	6.54E-003
dme-mir-279-3p	0.970	14.073	1.06E-003	7.38E-003
dme-mir-303-3p	-2.962	4.695	1.13E-003	7.74E-003
dme-mir-2a-1-3p	-1.522	6.794	1.31E-003	8.83E-003
dme-mir-275-3p	1.316	10.182	1.40E-003	9.28E-003
dme-mir-306-3p	1.278	8.378	1.60E-003	1.05E-002
dme-mir-274-5p	-0.935	11.181	2.61E-003	1.67E-002
dme-mir-965-5p	1.764	6.145	2.75E-003	1.74E-002
dme-mir-964-3p	-5.959	4.246	3.08E-003	1.92E-002
dme-mir-282-5p	0.799	12.437	3.73E-003	2.30E-002

dme-mir-316-3p	1.563	6.113	3.87E-003	2.35E-002
dme-mir-13b-1-3p	2.194	5.071	5.00E-003	2.99E-002
dme-mir-958-5p	0.997	8.647	5.16E-003	3.01E-002
dme-mir-962-3p	-2.583	5.319	5.16E-003	3.01E-002
dme-mir-286-3p	2.297	5.081	5.50E-003	3.16E-002
dme-mir-iab-8-3p	-5.794	4.078	6.41E-003	3.64E-002
dme-mir-34-3p	-0.732	12.786	7.83E-003	4.39E-002
dme-mir-252-5p	-0.717	14.342	8.00E-003	4.42E-002
dme-mir-375-5p	-1.249	6.684	8.21E-003	4.49E-002
dme-mir-993-5p	-1.115	6.873	9.48E-003	5.11E-002
dme-mir-974-5p	-5.684	4.049	9.73E-003	5.19E-002
dme-mir-308-3p	0.858	9.915	1.09E-002	5.75E-002
dme-mir-283-5p	0.880	7.975	1.15E-002	5.97E-002
dme-mir-285-3p	0.902	9.459	1.16E-002	5.97E-002
dme-mir-309-3p	5.872	3.863	1.19E-002	6.03E-002
dme-mir-956-5p	1.394	6.851	1.25E-002	6.27E-002
dme-mir-7-5p	0.725	10.766	1.37E-002	6.82E-002
dme-mir-1017-3p	-1.280	6.329	1.42E-002	6.96E-002
dme-mir-87-5p	2.211	4.783	1.52E-002	7.38E-002
dme-mir-2498-3p	-5.654	4.074	1.70E-002	8.16E-002
dme-mir-281-1-5p	-0.996	7.832	1.74E-002	8.27E-002
dme-mir-276a-3p	-0.670	15.869	1.79E-002	8.41E-002
dme-mir-1004-3p	-1.229	6.568	1.82E-002	8.44E-002
dme-mir-4976-5p	-2.267	5.055	1.83E-002	8.44E-002
dme-mir-2494-3p	0.909	7.714	1.92E-002	8.75E-002
dme-mir-988-3p	0.693	12.759	2.08E-002	9.35E-002
dme-mir-193-3p	1.279	6.938	2.10E-002	9.37E-002
dme-mir-277-3p	-0.601	16.293	2.38E-002	1.05E-001
dme-mir-2b-1-3p	0.836	7.359	2.41E-002	1.05E-001
dme-mir-317-5p	-0.709	11.626	2.50E-002	1.08E-001
dme-mir-219-5p	-0.847	8.343	2.93E-002	1.26E-001
dme-mir-979-3p	-5.312	3.849	3.07E-002	1.30E-001
dme-mir-13a-3p	-0.978	8.253	3.30E-002	1.39E-001
dme-mir-2a-2-3p	0.586	10.925	4.26E-002	1.77E-001
dme-mir-317-3p	-0.529	14.781	4.34E-002	1.79E-001
dme-mir-375-3p	-0.693	11.019	4.52E-002	1.85E-001
dme-mir-iab-8-5p	-1.774	5.192	4.62E-002	1.87E-001

dme-mir-124-3p	-0.559	13.244	4.95E-002	1.96E-001
dme-mir-284-3p	-0.562	10.762	4.95E-002	1.96E-001
dme-mir-992-5p	-2.952	4.037	4.99E-002	1.96E-001
dme-mir-1012-3p	0.954	7.169	5.07E-002	1.98E-001
dme-mir-7-3p	1.363	5.194	5.32E-002	2.06E-001
dme-mir-2b-2-5p	0.707	7.893	5.58E-002	2.13E-001
dme-mir-997-3p	-4.879	3.525	5.59E-002	2.13E-001
dme-mir-184-5p	0.988	7.461	5.68E-002	2.14E-001
dme-mir-14-5p	0.837	8.030	5.82E-002	2.17E-001
dme-mir-133-3p	-0.536	11.214	6.06E-002	2.25E-001
dme-mir-1010-5p	0.907	6.017	6.39E-002	2.35E-001
dme-mir-1014-5p	-1.285	4.933	6.64E-002	2.42E-001
dme-mir-4977-3p	4.867	3.284	7.58E-002	2.74E-001
dme-mir-962-5p	-1.410	5.431	8.74E-002	3.10E-001
dme-mir-973-3p	-4.705	3.494	8.76E-002	3.10E-001
dme-mir-983-2-5p	-2.980	3.823	8.79E-002	3.10E-001
dme-mir-304-3p	-1.192	5.682	8.99E-002	3.14E-001
dme-mir-965-3p	0.657	8.452	9.11E-002	3.16E-001
dme-mir-31b-3p	-0.584	7.966	9.48E-002	3.26E-001
dme-mir-125-5p	-0.469	13.484	9.93E-002	3.39E-001
dme-mir-1-5p	-0.593	9.885	1.04E-001	3.52E-001
dme-mir-8-3p	-0.440	15.801	1.05E-001	3.52E-001
dme-mir-929-3p	-0.676	7.016	1.11E-001	3.71E-001
dme-mir-305-3p	0.466	10.080	1.23E-001	4.07E-001
dme-mir-986-5p	-0.598	8.637	1.27E-001	4.18E-001
dme-mir-13b-2-3p	1.647	4.606	1.35E-001	4.41E-001
dme-mir-1-3p	-0.393	16.561	1.39E-001	4.48E-001
dme-mir-979-5p	-4.329	3.318	1.44E-001	4.64E-001
dme-let-7-3p	0.480	7.762	1.56E-001	4.98E-001
dme-mir-137-3p	-0.635	7.151	1.59E-001	5.03E-001
dme-mir-1003-3p	0.480	9.810	1.65E-001	5.19E-001
dme-mir-929-5p	-1.042	5.024	1.67E-001	5.20E-001
dme-mir-284-5p	-0.448	9.061	1.68E-001	5.20E-001
dme-mir-982-3p	-4.386	3.251	1.71E-001	5.20E-001
dme-mir-2535b-3p	-1.537	3.876	1.71E-001	5.20E-001
dme-mir-2494-5p	1.558	4.355	1.72E-001	5.20E-001

dme-mir-966-3p	1.335	3.870	1.72E-001	5.20E-001
dme-mir-932-5p	-0.439	8.538	1.76E-001	5.25E-001
dme-mir-275-5p	0.500	8.302	1.77E-001	5.25E-001
dme-mir-31a-3p	-0.751	7.044	1.79E-001	5.28E-001
dme-mir-999-3p	-0.412	9.605	1.82E-001	5.31E-001
dme-mir-125-3p	-0.843	5.955	1.82E-001	5.31E-001
dme-mir-190-5p	-0.421	8.769	1.88E-001	5.44E-001
dme-mir-3-3p	0.887	4.601	2.06E-001	5.91E-001
dme-mir-193-5p	1.209	4.255	2.14E-001	6.10E-001
dme-mir-210-5p	0.540	5.982	2.17E-001	6.15E-001
dme-mir-955-5p	-1.264	5.423	2.28E-001	6.44E-001
dme-mir-1009-3p	0.773	5.594	2.31E-001	6.47E-001
dme-mir-1001-5p	-0.500	6.938	2.38E-001	6.61E-001
dme-mir-210-3p	0.380	9.939	2.44E-001	6.75E-001
dme-mir-1003-5p	1.004	4.007	2.48E-001	6.81E-001
dme-mir-304-5p	-0.343	9.620	2.62E-001	7.17E-001
dme-mir-983-1-3p	-2.539	3.698	2.69E-001	7.22E-001
dme-mir-281-2-5p	-0.382	10.373	2.69E-001	7.22E-001
dme-mir-iab-4-5p	-0.512	6.991	2.69E-001	7.22E-001
dme-mir-1004-5p	2.438	3.251	2.84E-001	7.57E-001
dme-mir-4968-5p	-1.016	3.555	2.89E-001	7.66E-001
dme-mir-1014-3p	-0.696	5.306	2.93E-001	7.70E-001
dme-mir-969-5p	-0.808	4.324	2.98E-001	7.80E-001
dme-mir-282-3p	0.301	10.493	3.01E-001	7.83E-001
dme-mir-33-3p	0.406	7.241	3.17E-001	8.15E-001
dme-mir-11-5p	0.570	6.171	3.17E-001	8.15E-001
dme-mir-307a-5p	-0.490	7.348	3.29E-001	8.41E-001
dme-mir-1005-3p	1.008	4.222	3.32E-001	8.45E-001
dme-mir-263b-5p	0.360	9.449	3.47E-001	8.76E-001
dme-mir-190-3p	0.736	5.575	3.50E-001	8.78E-001
dme-mir-31b-5p	-0.261	10.128	3.71E-001	9.24E-001
dme-mir-276b-3p	-0.344	9.111	3.72E-001	9.24E-001
dme-mir-87-3p	-0.275	10.177	3.85E-001	9.50E-001
dme-mir-11-3p	0.289	13.773	3.87E-001	9.51E-001
dme-bantam-3p	0.241	12.647	3.99E-001	9.73E-001
dme-mir-10-5p	0.268	9.198	4.01E-001	9.73E-001
dme-mir-2499-3p	-1.999	3.588	4.15E-001	1.00E+000

dme-mir-34-5p	-0.222	16.769	4.18E-001	1.00E+000
dme-mir-10-3p	-0.220	11.764	4.56E-001	1.00E+000
dme-mir-957-3p	-0.363	8.177	4.62E-001	1.00E+000
dme-mir-1000-5p	-0.396	7.494	4.66E-001	1.00E+000
dme-mir-9a-3p	-0.416	7.165	4.79E-001	1.00E+000
dme-mir-1015-3p	0.272	7.698	4.84E-001	1.00E+000
dme-mir-954-5p	-0.422	6.558	4.98E-001	1.00E+000
dme-mir-4913-3p	3.620	2.885	5.01E-001	1.00E+000
dme-mir-315-5p	0.655	4.274	5.02E-001	1.00E+000
dme-mir-983-1-5p	3.629	2.885	5.03E-001	1.00E+000
dme-mir-2498-5p	-2.072	2.726	5.04E-001	1.00E+000
dme-mir-2500-3p	-3.396	2.996	5.05E-001	1.00E+000
dme-mir-4975-5p	-3.405	2.996	5.07E-001	1.00E+000
dme-mir-972-5p	-3.408	2.996	5.07E-001	1.00E+000
dme-mir-8-5p	-0.190	12.385	5.07E-001	1.00E+000
dme-mir-1016-3p	-2.099	2.726	5.08E-001	1.00E+000
dme-mir-4958-3p	0.776	2.868	5.12E-001	1.00E+000
dme-mir-1007-5p	-2.145	2.726	5.13E-001	1.00E+000
dme-mir-1006-3p	-0.230	6.924	5.13E-001	1.00E+000
dme-mir-3641-3p	0.794	2.868	5.15E-001	1.00E+000
dme-mir-976-5p	-3.678	3.081	5.19E-001	1.00E+000
dme-mir-974-3p	-4.198	3.287	5.19E-001	1.00E+000
dme-mir-4942-3p	-2.204	2.726	5.20E-001	1.00E+000
dme-mir-5-5p	2.069	3.129	5.21E-001	1.00E+000
dme-mir-3645-5p	-2.218	2.726	5.22E-001	1.00E+000
dme-mir-285-5p	-0.371	4.360	5.25E-001	1.00E+000
dme-mir-971-3p	-0.290	4.968	5.28E-001	1.00E+000
dme-mir-3643-5p	2.896	2.726	5.28E-001	1.00E+000
dme-mir-14-3p	-0.178	17.074	5.30E-001	1.00E+000
dme-mir-2497-5p	2.912	2.726	5.31E-001	1.00E+000
dme-mir-1002-5p	2.920	2.726	5.32E-001	1.00E+000
dme-mir-2279-3p	2.929	2.726	5.34E-001	1.00E+000
dme-mir-927-3p	0.992	3.916	5.37E-001	1.00E+000
dme-mir-2279-5p	0.780	4.587	5.42E-001	1.00E+000
dme-mir-1010-3p	-0.231	7.448	5.46E-001	1.00E+000
dme-mir-124-5p	1.200	3.900	5.64E-001	1.00E+000
dme-mir-2a-2-5p	0.254	11.790	5.64E-001	1.00E+000

dme-mir-iab-4-3p	-0.408	5.109	5.66E-001	1.00E+000
dme-mir-307a-3p	0.172	10.213	5.72E-001	1.00E+000
dme-mir-33-5p	0.173	10.201	5.77E-001	1.00E+000
dme-mir-4969-5p	-1.306	3.793	5.87E-001	1.00E+000
dme-mir-276b-5p	0.786	5.515	6.06E-001	1.00E+000
dme-mir-954-3p	0.683	4.895	6.07E-001	1.00E+000
dme-mir-987-5p	0.147	8.683	6.14E-001	1.00E+000
dme-mir-980-3p	0.137	8.318	6.16E-001	1.00E+000
dme-mir-2501-5p	0.386	3.236	6.53E-001	1.00E+000
dme-mir-987-3p	-1.417	3.333	6.54E-001	1.00E+000
dme-mir-4-3p	0.907	3.349	6.54E-001	1.00E+000
dme-mir-137-5p	0.393	3.236	6.54E-001	1.00E+000
dme-mir-133-5p	-0.642	4.629	6.55E-001	1.00E+000
dme-mir-984-3p	-1.999	3.633	6.58E-001	1.00E+000
dme-mir-1008-3p	0.200	7.486	6.58E-001	1.00E+000
dme-mir-970-3p	0.134	10.138	6.74E-001	1.00E+000
dme-mir-278-5p	-0.234	7.524	6.80E-001	1.00E+000
dme-mir-2c-3p	-0.570	5.826	7.04E-001	1.00E+000
dme-mir-1006-5p	0.368	3.692	7.15E-001	1.00E+000
dme-bantam-5p	0.099	9.927	7.21E-001	1.00E+000
dme-mir-927-5p	-0.150	7.535	7.22E-001	1.00E+000
dme-mir-966-5p	0.442	4.987	7.32E-001	1.00E+000
dme-mir-252-3p	0.148	9.108	7.62E-001	1.00E+000
dme-mir-4955-3p	0.152	3.963	7.63E-001	1.00E+000
dme-mir-988-5p	0.166	8.098	7.64E-001	1.00E+000
dme-mir-314-3p	0.100	10.429	7.65E-001	1.00E+000
dme-mir-281-2-3p	0.417	5.077	7.68E-001	1.00E+000
dme-mir-307b-3p	0.090	3.906	7.76E-001	1.00E+000
dme-let-7-5p	-0.084	14.256	7.79E-001	1.00E+000
dme-mir-31a-5p	-0.073	12.176	7.94E-001	1.00E+000
dme-mir-263a-3p	0.025	6.380	8.02E-001	1.00E+000
dme-mir-283-3p	0.224	5.855	8.12E-001	1.00E+000
dme-mir-981-3p	0.054	9.396	8.19E-001	1.00E+000
dme-mir-990-5p	0.034	4.134	8.20E-001	1.00E+000
dme-mir-9a-5p	0.064	12.502	8.30E-001	1.00E+000
dme-mir-316-5p	-0.072	10.846	8.44E-001	1.00E+000
dme-mir-1013-3p	0.400	4.897	8.63E-001	1.00E+000

dme-mir-12-3p	-0.221	6.446	8.65E-001	1.00E+000
dme-mir-986-3p	-0.112	5.281	8.76E-001	1.00E+000
dme-mir-100-5p	0.059	8.829	8.77E-001	1.00E+000
dme-mir-263a-5p	0.047	12.463	8.84E-001	1.00E+000
dme-mir-970-5p	0.040	5.669	9.05E-001	1.00E+000
dme-mir-2b-2-3p	-0.037	5.887	9.29E-001	1.00E+000
dme-mir-305-5p	-0.023	13.881	9.29E-001	1.00E+000
dme-mir-958-3p	-0.024	13.542	9.31E-001	1.00E+000
dme-mir-998-5p	-0.013	8.571	9.79E-001	1.00E+000
dme-mir-956-3p	0.012	11.570	9.79E-001	1.00E+000
dme-mir-277-5p	0.002	11.789	9.90E-001	1.00E+000
dme-mir-278-3p	0.001	13.189	9.96E-001	1.00E+000
dme-mir-286-5p	1.549	2.997	1.00E+000	1.00E+000
dme-mir-1015-5p	-0.527	4.105	1.00E+000	1.00E+000
dme-mir-4919-3p	0.221	3.268	1.00E+000	1.00E+000
dme-mir-2c-5p	-0.090	5.187	1.00E+000	1.00E+000
dme-mir-281-1-3p	0.379	4.848	1.00E+000	1.00E+000
dme-mir-4949-5p	-1.479	3.394	1.00E+000	1.00E+000
dme-mir-1007-3p	-0.287	4.818	1.00E+000	1.00E+000
dme-mir-4918-3p	3.614	2.869	1.00E+000	1.00E+000
dme-mir-2497-3p	3.613	2.869	1.00E+000	1.00E+000
dme-mir-968-5p	3.612	2.869	1.00E+000	1.00E+000
dme-mir-963-3p	-3.366	2.964	1.00E+000	1.00E+000
dme-mir-4943-3p	-3.366	2.964	1.00E+000	1.00E+000
dme-mir-4961-5p	-3.366	2.964	1.00E+000	1.00E+000
dme-mir-4983-3p	-3.364	2.964	1.00E+000	1.00E+000
dme-mir-4952-5p	-2.845	2.836	1.00E+000	1.00E+000
dme-mir-4981-5p	-2.842	2.836	1.00E+000	1.00E+000
dme-mir-4939-3p	-2.841	2.836	1.00E+000	1.00E+000
dme-mir-990-3p	-2.838	2.836	1.00E+000	1.00E+000
dme-mir-4981-3p	-2.837	2.836	1.00E+000	1.00E+000
dme-mir-975-3p	-2.835	2.836	1.00E+000	1.00E+000
dme-mir-6-2-5p	2.727	2.710	1.00E+000	1.00E+000
dme-mir-100-3p	2.727	2.710	1.00E+000	1.00E+000
dme-mir-932-3p	2.727	2.710	1.00E+000	1.00E+000
dme-mir-1005-5p	2.727	2.710	1.00E+000	1.00E+000
dme-mir-1016-5p	2.727	2.710	1.00E+000	1.00E+000

dme-mir-2493-5p	2.727	2.710	1.00E+000	1.00E+000
dme-mir-3645-3p	2.727	2.710	1.00E+000	1.00E+000
dme-mir-4909-3p	2.727	2.710	1.00E+000	1.00E+000
dme-mir-4913-5p	2.727	2.710	1.00E+000	1.00E+000
dme-mir-4917-3p	2.727	2.710	1.00E+000	1.00E+000
dme-mir-4957-3p	2.727	2.710	1.00E+000	1.00E+000
dme-mir-4962-3p	2.727	2.710	1.00E+000	1.00E+000
dme-mir-4979-3p	2.727	2.710	1.00E+000	1.00E+000
dme-mir-4983-5p	2.727	2.710	1.00E+000	1.00E+000
dme-mir-4-5p	-2.037	2.693	1.00E+000	1.00E+000
dme-mir-6-1-5p	-2.037	2.693	1.00E+000	1.00E+000
dme-mir-13a-5p	-2.037	2.693	1.00E+000	1.00E+000
dme-mir-219-3p	-2.037	2.693	1.00E+000	1.00E+000
dme-mir-955-3p	-2.037	2.693	1.00E+000	1.00E+000
dme-mir-967-5p	-2.037	2.693	1.00E+000	1.00E+000
dme-mir-991-5p	-2.037	2.693	1.00E+000	1.00E+000
dme-mir-2496-5p	-2.037	2.693	1.00E+000	1.00E+000
dme-mir-2499-5p	-2.037	2.693	1.00E+000	1.00E+000
dme-mir-3642-5p	-2.037	2.693	1.00E+000	1.00E+000
dme-mir-4914-5p	-2.037	2.693	1.00E+000	1.00E+000
dme-mir-4915-3p	-2.037	2.693	1.00E+000	1.00E+000
dme-mir-4947-5p	-2.037	2.693	1.00E+000	1.00E+000
dme-mir-4950-3p	-2.037	2.693	1.00E+000	1.00E+000
dme-mir-4967-3p	-2.037	2.693	1.00E+000	1.00E+000
dme-mir-4969-3p	-2.037	2.693	1.00E+000	1.00E+000
dme-mir-4972-5p	-2.037	2.693	1.00E+000	1.00E+000
dme-mir-4976-3p	-2.037	2.693	1.00E+000	1.00E+000
dme-mir-4977-5p	-2.037	2.693	1.00E+000	1.00E+000
dme-mir-4980-3p	-2.037	2.693	1.00E+000	1.00E+000
dme-mir-4985-5p	-2.037	2.693	1.00E+000	1.00E+000
dme-mir-4986-3p	-2.037	2.693	1.00E+000	1.00E+000
dme-mir-274-3p	-1.192	3.863	1.00E+000	1.00E+000
dme-mir-2489-3p	-1.091	3.318	1.00E+000	1.00E+000
dme-mir-2500-5p	1.077	3.267	1.00E+000	1.00E+000
dme-mir-315-3p	-0.976	3.204	1.00E+000	1.00E+000
dme-mir-983-2-3p	-0.971	3.204	1.00E+000	1.00E+000
dme-mir-1001-3p	-0.849	3.619	1.00E+000	1.00E+000

dme-mir-4964-3p	0.690	2.852	1.00E+000	1.00E+000
dme-mir-2495-5p	0.690	2.852	1.00E+000	1.00E+000
dme-mir-985-5p	0.690	2.852	1.00E+000	1.00E+000
dme-mir-4919-5p	-0.664	3.589	1.00E+000	1.00E+000
dme-mir-5-3p	-0.633	3.145	1.00E+000	1.00E+000
dme-mir-999-5p	0.623	4.321	1.00E+000	1.00E+000
dme-mir-2493-3p	-0.612	3.097	1.00E+000	1.00E+000
dme-mir-307b-5p	-0.610	3.097	1.00E+000	1.00E+000
dme-mir-263b-3p	-0.481	3.824	1.00E+000	1.00E+000
dme-mir-1000-3p	-0.427	3.113	1.00E+000	1.00E+000
dme-mir-2b-1-5p	0.414	3.424	1.00E+000	1.00E+000
dme-mir-980-5p	0.321	4.180	1.00E+000	1.00E+000
dme-mir-4968-3p	-0.298	3.013	1.00E+000	1.00E+000
dme-mir-4951-3p	-0.276	3.920	1.00E+000	1.00E+000
dme-mir-4949-3p	-0.132	3.634	1.00E+000	1.00E+000
dme-mir-2535b-5p	-0.122	2.980	1.00E+000	1.00E+000
dme-mir-4940-5p	-0.119	2.980	1.00E+000	1.00E+000
dme-mir-4951-5p	0.117	4.232	1.00E+000	1.00E+000
dme-mir-3641-5p	-0.114	3.318	1.00E+000	1.00E+000
dme-mir-276a-5p	0.079	4.367	1.00E+000	1.00E+000
dme-mir-1012-5p	0.067	7.234	1.00E+000	1.00E+000
dme-mir-3-5p	0.000	2.532	1.00E+000	1.00E+000
dme-mir-6-3-5p	0.000	2.532	1.00E+000	1.00E+000
dme-mir-6-3-3p	0.000	2.532	1.00E+000	1.00E+000
dme-mir-13b-1-5p	0.000	2.532	1.00E+000	1.00E+000
dme-mir-280-5p	0.000	2.532	1.00E+000	1.00E+000
dme-mir-287-3p	0.000	2.532	1.00E+000	1.00E+000
dme-mir-288-3p	0.000	2.532	1.00E+000	1.00E+000
dme-mir-309-5p	0.000	2.532	1.00E+000	1.00E+000
dme-mir-957-5p	0.000	2.532	1.00E+000	1.00E+000
dme-mir-967-3p	0.000	2.532	1.00E+000	1.00E+000
dme-mir-968-3p	0.000	2.532	1.00E+000	1.00E+000
dme-mir-969-3p	0.000	2.532	1.00E+000	1.00E+000
dme-mir-971-5p	0.000	2.532	1.00E+000	1.00E+000
dme-mir-981-5p	0.000	2.532	1.00E+000	1.00E+000
dme-mir-1002-3p	0.000	2.532	1.00E+000	1.00E+000

dme-mir-1008-5p	0.000	2.532	1.00E+000	1.00E+000
dme-mir-1009-5p	0.000	2.532	1.00E+000	1.00E+000
dme-mir-1011-3p	0.000	2.532	1.00E+000	1.00E+000
dme-mir-1013-5p	0.000	2.532	1.00E+000	1.00E+000
dme-mir-1017-5p	0.000	2.532	1.00E+000	1.00E+000
dme-mir-2280-5p	0.000	2.532	1.00E+000	1.00E+000
dme-mir-2280-3p	0.000	2.532	1.00E+000	1.00E+000
dme-mir-2281-5p	0.000	2.532	1.00E+000	1.00E+000
dme-mir-2281-3p	0.000	2.532	1.00E+000	1.00E+000
dme-mir-2282-3p	0.000	2.532	1.00E+000	1.00E+000
dme-mir-2283-5p	0.000	2.532	1.00E+000	1.00E+000
dme-mir-2283-3p	0.000	2.532	1.00E+000	1.00E+000
dme-mir-2490-5p	0.000	2.532	1.00E+000	1.00E+000
dme-mir-2491-5p	0.000	2.532	1.00E+000	1.00E+000
dme-mir-2492-3p	0.000	2.532	1.00E+000	1.00E+000
dme-mir-2501-3p	0.000	2.532	1.00E+000	1.00E+000
dme-mir-3642-3p	0.000	2.532	1.00E+000	1.00E+000
dme-mir-3644-5p	0.000	2.532	1.00E+000	1.00E+000
dme-mir-3644-3p	0.000	2.532	1.00E+000	1.00E+000
dme-mir-4908-5p	0.000	2.532	1.00E+000	1.00E+000
dme-mir-4908-3p	0.000	2.532	1.00E+000	1.00E+000
dme-mir-4909-5p	0.000	2.532	1.00E+000	1.00E+000
dme-mir-4910-5p	0.000	2.532	1.00E+000	1.00E+000
dme-mir-4911-3p	0.000	2.532	1.00E+000	1.00E+000
dme-mir-4912-5p	0.000	2.532	1.00E+000	1.00E+000
dme-mir-4914-3p	0.000	2.532	1.00E+000	1.00E+000
dme-mir-4915-5p	0.000	2.532	1.00E+000	1.00E+000
dme-mir-4917-5p	0.000	2.532	1.00E+000	1.00E+000
dme-mir-4918-5p	0.000	2.532	1.00E+000	1.00E+000
dme-mir-4940-3p	0.000	2.532	1.00E+000	1.00E+000
dme-mir-4941-5p	0.000	2.532	1.00E+000	1.00E+000
dme-mir-4941-3p	0.000	2.532	1.00E+000	1.00E+000
dme-mir-4945-5p	0.000	2.532	1.00E+000	1.00E+000
dme-mir-4947-3p	0.000	2.532	1.00E+000	1.00E+000
dme-mir-4948-5p	0.000	2.532	1.00E+000	1.00E+000
dme-mir-4948-3p	0.000	2.532	1.00E+000	1.00E+000
dme-mir-4950-5p	0.000	2.532	1.00E+000	1.00E+000

dme-mir-4952-3p	0.000	2.532	1.00E+000	1.00E+000
dme-mir-4953-3p	0.000	2.532	1.00E+000	1.00E+000
dme-mir-4954-5p	0.000	2.532	1.00E+000	1.00E+000
dme-mir-4954-3p	0.000	2.532	1.00E+000	1.00E+000
dme-mir-4955-5p	0.000	2.532	1.00E+000	1.00E+000
dme-mir-4956-5p	0.000	2.532	1.00E+000	1.00E+000
dme-mir-4956-3p	0.000	2.532	1.00E+000	1.00E+000
dme-mir-4957-5p	0.000	2.532	1.00E+000	1.00E+000
dme-mir-4958-5p	0.000	2.532	1.00E+000	1.00E+000
dme-mir-4959-5p	0.000	2.532	1.00E+000	1.00E+000
dme-mir-4960-5p	0.000	2.532	1.00E+000	1.00E+000
dme-mir-4960-3p	0.000	2.532	1.00E+000	1.00E+000
dme-mir-4961-3p	0.000	2.532	1.00E+000	1.00E+000
dme-mir-4962-5p	0.000	2.532	1.00E+000	1.00E+000
dme-mir-4963-5p	0.000	2.532	1.00E+000	1.00E+000
dme-mir-4963-3p	0.000	2.532	1.00E+000	1.00E+000
dme-mir-4965-5p	0.000	2.532	1.00E+000	1.00E+000
dme-mir-4965-3p	0.000	2.532	1.00E+000	1.00E+000
dme-mir-4970-5p	0.000	2.532	1.00E+000	1.00E+000
dme-mir-4970-3p	0.000	2.532	1.00E+000	1.00E+000
dme-mir-4971-5p	0.000	2.532	1.00E+000	1.00E+000
dme-mir-4971-3p	0.000	2.532	1.00E+000	1.00E+000
dme-mir-4972-3p	0.000	2.532	1.00E+000	1.00E+000
dme-mir-4973-5p	0.000	2.532	1.00E+000	1.00E+000
dme-mir-4973-3p	0.000	2.532	1.00E+000	1.00E+000
dme-mir-4974-5p	0.000	2.532	1.00E+000	1.00E+000
dme-mir-4974-3p	0.000	2.532	1.00E+000	1.00E+000
dme-mir-4975-3p	0.000	2.532	1.00E+000	1.00E+000
dme-mir-4982-5p	0.000	2.532	1.00E+000	1.00E+000
dme-mir-4982-3p	0.000	2.532	1.00E+000	1.00E+000
dme-mir-4984-5p	0.000	2.532	1.00E+000	1.00E+000
dme-mir-4984-3p	0.000	2.532	1.00E+000	1.00E+000
dme-mir-4986-5p	0.000	2.532	1.00E+000	1.00E+000
dme-mir-4987-5p	0.000	2.532	1.00E+000	1.00E+000
dme-mir-4987-3p	0.000	2.532	1.00E+000	1.00E+000
