# Supplementary material for: Sex-biased expression of microRNAs in Drosophila melanogaster
Source: Open Biol. 2014 Apr 2;4(4):140024. doi: 10.1098/rsob.140024 (PMC4043116; doi:10.1098/rsob.140024)
Supplement: Supplementary Table 2 [file rsob140024supp2.pdf]

Supplementary Table 2. Difference in fold change between all targeted genes and genes targeted by recently evolved male-biased microRNAs.

	Target Prediction Algorithm					
MicroRNA	miRanda		DianaT		TargetScan	
	logFC*	p-value**	logFC*	p-value**	logFC*	p-value**
ALL	0.229	-	0.053	-	0.147	-
mir-985-3p	0.339	0.045	-0.020	0.002	0.000	0.000
mir-997-5p	0.618	0.001	0.249	0.039	0.237	0.002
mir-991-3p	0.797	0.296	0.020	0.437	0.034	0.005
mir-992-3p	0.366	0.812	0.008	0.266	0.006	0.131
mir-982-5p	0.739	0.047	0.000	0.358	0.124	0.002
mir-984-5p	0.299	0.708	0.096	0.161	0.258	0.018
mir-303-5p	0.346	0.476	0.135	0.417	0.126	0.090

* logFC: log2 fold-change male/female expression bias
** p-value: significance in Kolmogorov-Smirnov test for distribution differences
Highlighted in red: significant values for a False Discovery Rate of 5% (Benjamini and Hochberg 1995)
