# Supplementary material for: Sex-biased expression of microRNAs in Drosophila melanogaster
Source: Open Biol. 2014 Apr 2;4(4):140024. doi: 10.1098/rsob.140024 (PMC4043116; doi:10.1098/rsob.140024)
Supplement: Supplementary Table 3 [file rsob140024supp3.pdf]

Supplementary Table 3. Ratio of young/old targeted genes of male-biased evolutionarily young microRNAs.

	Target Prediction Algorithm					
MicroRNA	miRanda		DianaT		TargetScan	
	Y/O*	p-value**	Y/O*	p-value**	Y/O*	p-value**
Expected ratio	0.452	-	0.419	-	0.490	-
mir-985-3p	0.425	0.060	0.362	0.000	0.451	0.000
mir-997-5p	0.463	0.346	0.389	0.098	0.542	0.001
mir-991-3p	0.500	0.237	0.369	0.001	0.457	0.001
mir-992-3p	0.448	0.428	0.346	0.000	0.440	0.001
mir-982-5p	0.451	0.448	0.389	0.124	0.558	0.001
mir-984-5p	0.473	0.200	0.390	0.137	0.535	0.043
mir-303-5p	0.435	0.229	0.309	0.000	0.419	0.000

* Y/O: Ratio between young (Drosophila lineage) and old (conserved) genes among the targeted by the microRNA.
** p-value: For each microRNA a random distribution of Y/O ratios was generated by calculating the ration in each of 10,000 random pseudosamples keeping the number of total genes targeted. A p-value is calculated by estimating the smallest proportion of Y/O random ratio values above/below the expected ratio. Highlighted in red: significant values for a False Discovery Rate of 5% (Benjamini and Hochberg 1995)
